# miR-30b-5p targeting GRIN2A inhibits hippocampal damage in epilepsy

**DOI:** 10.1515/med-2023-0675

**Published:** 2023-03-30

**Authors:** Hu Zheng, Liuyang Wu, Huisheng Yuan

**Affiliations:** Department of Neurosurgery, Hubei Provincial Hospital of Integrated Chinese & Western Medicine, Wuhan 430015, Hubei, China; Department of Neurosurgery, Hubei Provincial Hospital of Integrated Chinese & Western Medicine, No. 11 Lingjiaohu Road, Jianghan District, Wuhan 430015, Hubei, China

**Keywords:** epilepsy, miRNA, targeting, proliferation

## Abstract

GRIN2A is associated with epilepsy (EP); however, its regulatory mechanism involving upstream miRNA (miR-30b-5p) has been overlooked. In this study, we aimed to identify the regulatory mechanism of the miR-30b-5p/GRIN2A axis in EP. Hippocampal neurons isolated from mice were incubated in magnesium-free medium for 48 h to establish an *in vitro* EP model. An *in vivo* model of EP was constructed by the intraperitoneal injection of atropine into mice. Nissl staining and hematoxylin and eosin staining were used to evaluate pathological injuries in the hippocampal CA1 regions of mice. The CCK8 assay confirmed that miR-30b-5p overexpression restored the suppressed proliferative capacity of hippocampal neurons exposed to magnesium-free conditions. Caspase-3 activity assay revealed that miR-30b-5p overexpression abrogated the increased apoptosis of hippocampal neurons under magnesium-free conditions. In an *in vivo* model of EP, miR-30b-5p overexpression reversed pathological injuries in the hippocampal CA1 regions of mice and abrogated the increased apoptosis in the EP mouse model. Luciferase assays and western blotting confirmed that miR-30b-5p targeted GRIN2A, thereby inhibiting GRIN2A expression. Overall, miR-30b-5p can protect against cell proliferation and attenuate apoptosis in hippocampal neurons under magnesium-free conditions by targeting GRIN2A.

## 1 Introduction

Epilepsy (EP) is a prevalent neurological disorder accompanied by transient cerebral dysfunction resulting from the aberrant synchronization of neuronal firing in the brain [1]. The main clinical manifestations of EP are repeated seizures and loss of consciousness and awareness, which causes a tremendous social and economic burden globally [2]. Global statistics have reported that 5 × 10^7^ patients were diagnosed with EP. Among them, 80% of patients were from the developing countries [3]. Although current anti-EP drugs can reduce the frequency of convulsive episodes, the disorder remains incurable [4]. Hippocampal CA3 and CA1 pyramidal neurons are the beginning sites of interictal epileptiform discharges, and the hippocampus is one of the critical sites of EP [2,5]. Hippocampal neurons exposed to magnesium-free conditions are often used to construct EP models *in vitro* [6,7]. Therefore, using magnesium-free conditions to treat hippocampal neurons to further decipher the biological progression of EP is a potential novel approach for EP diagnosis and intervention.

MicroRNAs (miRNAs) are transcripts of 20–25 nucleotides without a protein-coding ability. Compelling data demonstrate that miRNAs are critical players in different physiological and pathological cascades such as carcinogenesis and neurological disorders [8]. Aberrantly expressed miRNAs have been detected in EP and act as critical players in modulating neuronal apoptosis, glial hyperplasia and hypertrophy, neuroinflammation, and neuronal microarchitecture remodeling [9,10]. For example, an *in vivo* model of pentylenetetrazol-stimulated EP demonstrated that highly expressed miRNA-137 attenuates the frequency of seizures [11]. Furthermore, blockade of the NF-kB signaling pathway by microRNA-129-5p impedes autoimmune encephalomyelitis-related EP [12]. Elnady et al. found that miR-106b and miR-146a are robustly expressed in childhood EP and function as markers of this neurological disorder [13]. Recently, miR-30b-5p was found to be involved in drug-resistant EP [14]. However, little is known about whether and how miR-30b-5p modulates EP progression.

The *GRIN2A* gene is located on chromosome 16p13.2 consisting of 16 exons. This gene encodes a member of the glutamate-gated ion-channel protein family [15]. The encoded protein is an *N*-methyl-d-aspartate receptor subunit involved in long-term potentiation, an activity-dependent increase in the efficiency of synaptic transmission thought to underlie certain types of memory and learning [16]. Genetic mutations in *GRIN2A* are associated with epilepsy-aphasia spectrum disorders [17,18]. However, the mechanism through which GRIN2 influences EP remains unclear.

A previous investigation demonstrated that miRNAs exert their functions by recognizing the 3′-UTR target gene [19]. In this study, we employed a series of bioinformatics analyses and identified miR-30b-5p as a potential upstream regulator of GRIN2A, interfering with EP progression. A previous study reported that miR-30b-5p targeting *MYBL2* could suppress the proliferation and induce apoptosis of medulloblastoma cells [20]. The miR-30b-5p induced by NF-κB aggravates joint pain and loss of articular cartilage [21]. However, the role of miR-30b-5p in EP remains unknown. We first investigated the effects of miR-30b-5p overexpression on the proliferation and apoptosis of hippocampal neurons under magnesium-free conditions and in a pilocarpine-induced EP mouse model. Our investigation further revealed a potential mechanism of the miR-30b-5p/GRIN2A axis in EP progression. Our findings suggest that miR-30b-5p/GRIN2A plays an important role in EP progression.

## 2 Methods

### 2.1 Animal maintenance and housing

A total of 56 3-month-old mice weighing 20–25 g were obtained from the Laboratory Animal Center, Wuhan Institute of Virology, Chinese Academy of Sciences, Wuhan, China. The mice received normal feed and 12 h cycle of lighting with adequate food availability.


**Ethical approval:** The research related to animals’ use has been complied with national and institutional guidelines for animal care and was approved by the Institutional Animal Ethics Committee Review Board of the Hubei Provincial Hospital of Integrated Chinese & Western Medicine.

### 2.2 Hippocampal neurons isolation and culture

We separated hippocampal neurons from the brains of mice as instructed previously [22]. The separated hippocampal neurons were cultivated in RPMI-1640 medium containing 10% FBS and 1% penicillin/streptomycin at 37°C and 5% CO_2_. Morphological observations coupled with immunofluorescence staining were used to identify hippocampal neurons.

### 2.3 RNA pull-down assay

A Magnetic RNA-Protein Pull-Down Kit (Thermo Fisher Scientific, USA) was used to detect the interaction between GRIN2A and its potential target miRNAs. Briefly, isolated hippocampal neurons were treated with Pierce IP Lysis Buffer (Thermo Fisher Scientific, USA). After 10 min, cell lysates were collected and subjected to centrifugation. The prepared supernatants underwent co-incubation with magnetic beads (Thermo Fisher Scientific, USA) attached to biotinylated miRNA or miRNA-NC (Ribobio, China). RT-qPCR analysis was employed to determine GRIN2A mRNA levels in the pull-down proteins of the RNA–protein complexes.

### 2.4 Immunofluorescence staining

After cultivating them for 12 h, the hippocampal neurons were subjected to 10% goat serum for 1.5 h at room temperature before incubation with anti-MAP2 antibody (Abcam; 1:1,000) at 4°C. Next day, the cells were continuously detected using goat anti-rabbit IgG (Abcam; 1:5,000) for 24 h at 37°C for additional 1 h, followed by nuclear staining with DAPI (Sigma, USA) for 5 min. Finally, fluorescence activity was observed under a fluorescence microscope (Olympus CX23, Japan).

### 2.5 Cell transfection

miR-30b-5p mimic, mimic NC, pcDNA-3.1-GRIN2A (GRIN2A-overexpressing vector, GRIN2A OE), and the empty vector (pcDNA 3.1) were purchased from GenePharma (Shanghai, China). The synthesized mononucleotides and vectors were introduced into hippocampal neurons at 30% confluence with Lipo3000 and Opti-MEM (Thermo Fisher, USA) according to the manufacturer’s instructions.

### 2.6 Establishment of the *in vitro* EP model

Magnesium-free conditions are often used to induce EP *in vitro*, according to previous studies [6,7]. Therefore, isolated hippocampal neurons in the logarithmic growth phase were cultivated in magnesium-free media for 48 h to establish an *in vitro* EP model.

### 2.7 CCK8 assays

Hippocampal neurons in the different treatment groups were seeded in 96-well plates for 24 h. Then, 10 μL CCK8 reagent was added to the medium for the next 2 h of cultivation. Absorbance at 450 nm was measured using a microplate reader.

### 2.8 Assessment of caspase-3 activity

Caspase-3 activity in hippocampal neurons in different treatments was detected using a Caspase 3 Assay Kit (Merck, USA) following the manufacturer’s instructions. Briefly, 2 × 10^6^ cells were lysed in 100 µL ice-cold lysis buffer for 15 min. After microcentrifugation at 4℃, the supernatant was collected. Incubation with 2 mM Ac-DEVD-pNA was conducted for 1 h at 37℃. When the color changed, the A405 was read using a microplate ELISA reader.

### 2.9 Western blots

The hippocampal neurons were homogenized and sonicated in ice-cold lysis buffer for 30 min. The supernatant samples were diluted to assess protein concentration using a Pierce BCA kit (Thermo Fisher, USA). Similar amounts of proteins were loaded onto 10% SDS-PAGE and run at 200 V before electric transfer to PVDF membranes. After blocking with 10% non-fat milk, the proteins on the membrane were detected at 4°C using anti-orb29358 (orb571010, 1:1,000; Biorbyt, UK) and anti-GAPDH (orb555879, 1:1,000; Biorbyt, UK). After 24 h, secondary anti-antibodies (Cat#: orb27046, 1:1,000; Biorbyt, UK) were supplemented for co-incubation with the membrane for 1 h. Finally, Pierce ECL western blot substrate (Thermo Fisher, USA) was used for the staining and visualization of the target proteins.

### 2.10 RT-qPCR

Total RNA was isolated from hippocampal neurons using a MolPure Cell RNA Kit (Yeasen, China), according to the manufacturer’s instructions. A reverse transcription kit (Yeasen, China) was used for reverse transcription. The expression levels of miR-30b-5p and GRIN2A were determined using a Real-Time PCR kit (Thermo Fisher, USA) on a real-time fluorescence quantitative PCR system (ABI7500). Relative expression was calculated using the 2^−ΔΔCt^ method by normalizing U6 or GAPDH. Primers used are listed in Table 1.

**Table 1:** The primers used

Gene symbol	Primer sequence (5′…3′)
GRIN2A-F	TCAGTGCCTCCGTCTGGGTGA
GRIN2A-R	GCCCGTGGGGAGCTTCCCT
rno-miR-30b-5p-F	GGGCTGTAAACATCCTACAC
rno-miR-30b-5p-R	TGCGTGTCGTGGAGTC
GAPDH-F	TGATTCTACCCACGGCAAGTT
GAPDH-R	TGATGGGTTTCCCATTGATGA
U6-F	CGCTTCACGAATTTGCGTGTCAT
U6-R	GCTTCGGCAGCACATATACTAAAAT

### 2.11 Luciferase reporter assays

The putative binding sequence (wild-type, WT) of 3′-UTR GRIN2A with miR-30b-5p and the corresponding mutant (MUT) fragment were amplified and inserted into pGL3 Luciferase Reporter Vectors (Promega, USA). The resulting WT and MUT vectors were transfected into 60% confluent hippocampal neurons. At 48 h post-transfection, luminescence measurements were performed using a luciferase assay kit (Genomeditech, China).

### 2.12 Establishment of the mice model for EP

Pilocarpine (300 mg/kg) (Sigma, USA) was intraperitoneally injected into 30 healthy mice before receiving an injection of atropine (2.5 mL/kg). Epileptic seizures were recorded. After 2 h, diazepam (8.0 mg/kg) was intraperitoneally injected into the mice to terminate EP induction. When an epileptic seizure reached four grades and lasted for more than 2 h, status epilepticus (SE) occurred. After 2 months, an *in vivo* EP model was successfully established. Normal mice were intraperitoneally injected with an equal amount of normal saline.

### 2.13 Agomir injection

Fifteen mice were divided into three subgroups: SE, SE + agomir-NC, and SE + agromir-miR. Five mice that underwent intraperitoneal administration of equal amounts of normal saline were defined as the normal group. For the SE + agomir-NC and SE + agromir groups, agromir-miR-30b-5p and agomir-NC were chemically synthesized by GenePharma, China. Agomir and agomir-NC (30 nL) were microinjected into the hippocampal CA1 region of mice. Finally, the hippocampal CA1 region was separated from the euthanized mice.

### 2.14 Hematoxylin and eosin (HE) staining

Tissue slices of the hippocampal CA1 regions from different groups were prepared by Linmei Biotechnology Co., Ltd, China, according to the conventional method. Sections were dewaxed with xylene for 5 min and hydrated with different doses of ethanol for 5 min. After repeated rinsing with PBS, the sections were stained with hematoxylin for 2 min and washed in running water. Eosin was added, and the slides were covered for 5 s. After dehydration with ethanol, sections were coagulated at room temperature. Representative images were obtained under a microscope in the direction of the pathologist.

### 2.15 Nissl staining

The deparaffinized tissue slices were stained with 1% toluidine blue. After 1 h, the slices were treated with gradient alcohol (70–100%) for color separation and dehydration. Nissl-stained cells were imaged under a microscope.

### 2.16 TUNEL staining

TUNEL staining was performed to examine apoptosis in the tissues of the mouse hippocampal CA1 regions. After deparaffinization, the sections were treated with 3% hydrogen peroxide to block endogenous peroxidase activity before incubation with 20% fetal bovine serum and 3% bovine serum albumin. After 20 min, 50 µL of the TUNEL mixture was added to the sections, which were maintained in a humidified 37°C incubator. After 1 h, the sections were exposed to peroxidase conjugated anti-digoxin antibody at 37°C. After 20 min, the sections were rinsed with PBS and developed with diaminobenzidine before double staining with hematoxylin. TUNEL-positive cells in the CA1 region were observed using a 400× magnification light microscope (Leica DM750, USA).

### 2.17 Statistical analysis

Data are expressed as mean ± standard deviation and plotted using GraphPad Prism 9.0. Student’s *t*-test and one-way analysis of variance were used to determine significant differences between two groups or among multiple groups, respectively. Statistical significance was set at *P* < 0.05.

## 3 Results

### 3.1 Identification of key regulators in EP

**Figure 1:**
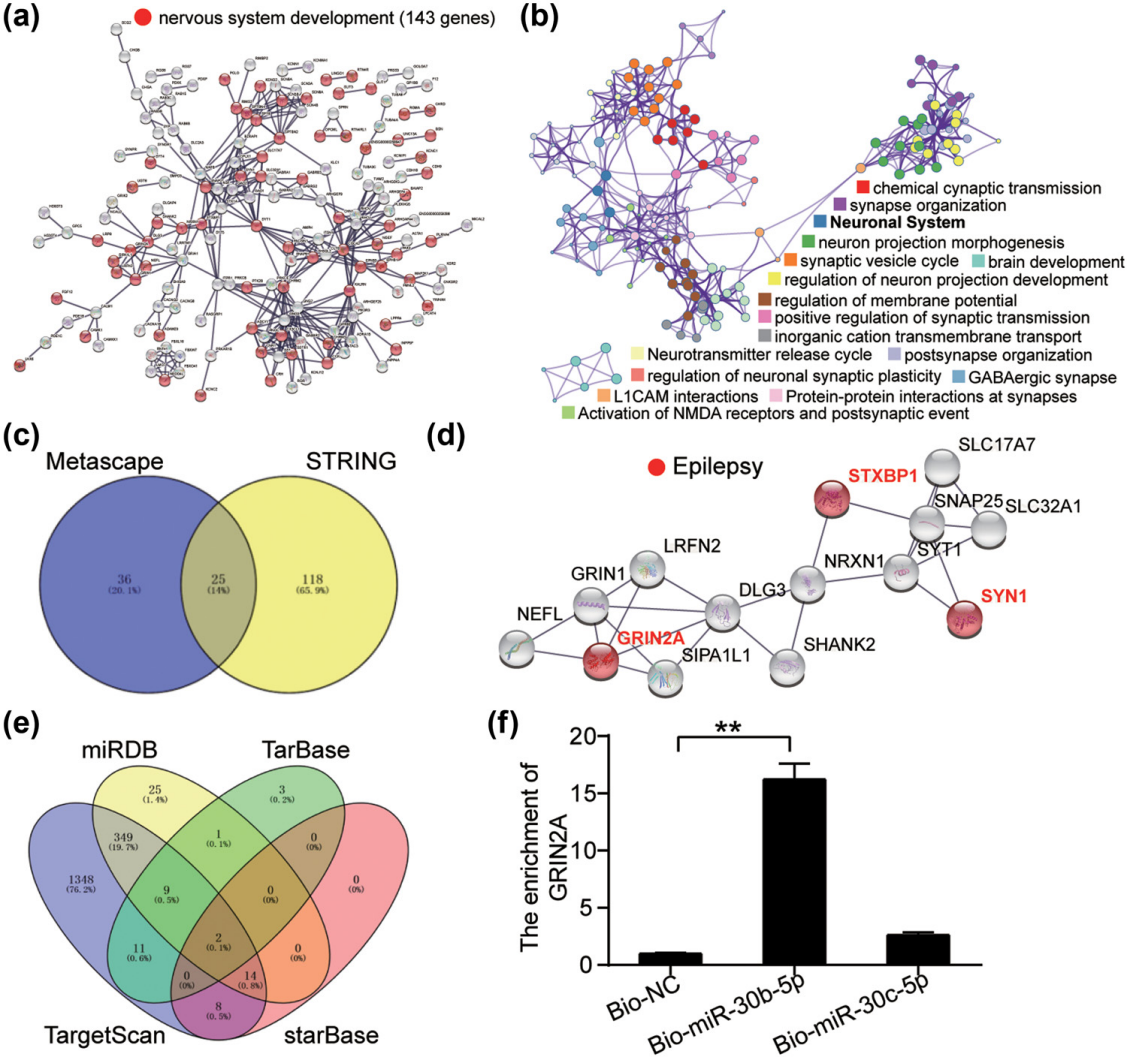
miR-30b-5p and GRIN2A were the key regulators in epilepsy. (a) STING was used for GO enrichment of 571 DEGs screened from GSE28674 with adj. *P* < 0.05. (b) Metascape was used for GO enrichment of 571 DEGs screened from GSE28674 with adj. *P* < 0.05. (c) Twenty-five genes involving nervous system development from STRING and neuronal system from Metascape overlapped. (d) STXBP1, GRIN2A, and SYN1 were enriched to be related to epilepsy. (e) Two miRNAs were predicted to bind to GRIN2A based on the four databases (miRDB, TarBase, TargetScan, and starBase). (f) Enrichment of GRIN2A was detected by RNA pull-down assay.

GSE28674 downloaded from GEO DataSets included the hippocampus with or without EP samples to screen for differentially expressed genes (DEGs). With adj. *P* < 0.05, 571 DEGs were screened and uploaded to STRING and Metascape for GO enrichment. STRING analysis showed that nervous system development involving 143 genes was the key GO process (Figure 1a), while Metascape found that the neuronal system involving 61 genes was the key GO process (Figure 1b). Then, 25 key genes were associated with nervous system development and neuronal system overlap (Figure 1c). After uploading the genes to *STRING*, *STXBP1*, *GRIN2A*, and *SYN1* among these 25 key genes were identified as the key genes involved in EP (Figure 1d). GRIN2A has been reported to be closely related to EP [23,24], but its upstream miRNAs that regulate its expression have not been explored in EP. To identify the upstream regions of GRIN2A, miRDB, TarBase, TargetScan, and starBase were used to predict miRNAs targeting GRIN2A. The miR-30b-5p and miR-30c-5p overlapped in all four databases (Figure 1e). RNA pull-down assay revealed that GRIN2A was enriched in hippocampal neurons transfected with bio-miR-30b-5p (Figure 1f).

### 3.2 miR-30b-5p overexpression abrogates the inhibitory effect exerted by magnesium-free condition on hippocampal neurons proliferation

**Figure 2:**
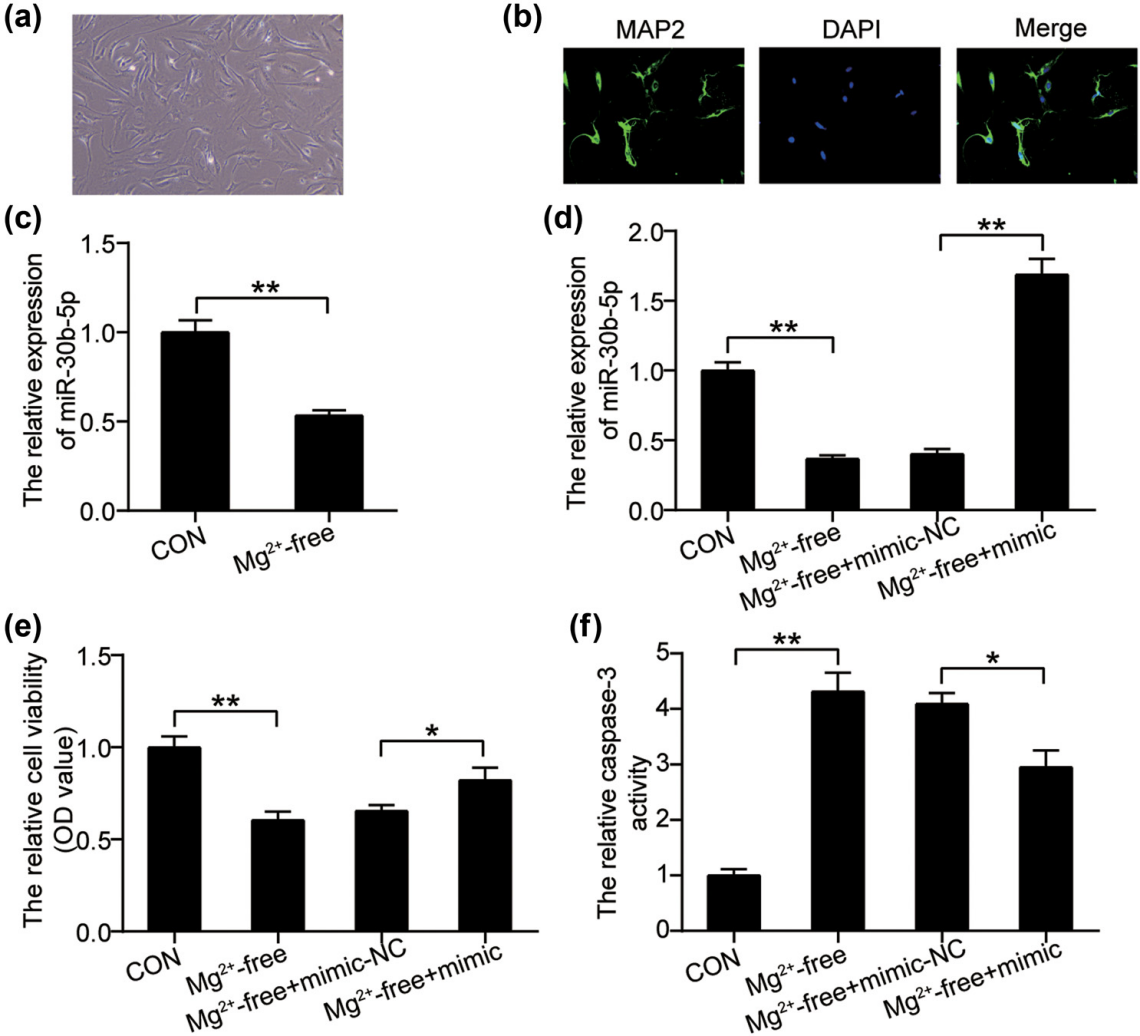
miR-30b-5p overexpression abrogates the inhibitory effect exerted by magnesium-free condition on hippocampal neurons proliferation. (a) Efficacy of isolation was observed by a light microscope at 200× magnification. (b) Efficiency of isolation was detected using immunofluorescence staining. The green fluorescence indicates MAP2, and the blue fluorescence indicates nuclear staining (DAPI). (c) RT-qPCR analysis of miR-30b-5p in hippocampal neurons in the presence or absence of free magnesium. (d) RT-qPCR analysis of miR-30b-5p in hippocampal neurons transfected with miR-30b-5p mimic or mimic NC in the presence or absence of free magnesium. (e) CCK8 assays determining the cell proliferation in hippocampal neurons transfected with miR-30b-5p mimic or mimic NC in the presence or absence of free magnesium. (f) Caspase-3 activity assessment in hippocampal neurons transfected with miR-30b-5p mimic or mimic NC in the presence or absence of free magnesium. *P < 0.05, **P < 0.001. NC, negative control.

Having verified the involvement of miR-30b-5p and GRIN2A in EP, we further investigated their functional roles in hippocampal neurons. We isolated hippocampal neurons from the brain tissue of normal rats. As shown in Figure 2a, morphological observations demonstrated that the hippocampal neurons were characterized by an elongated shape, which is a typical feature of hippocampal neurons. In parallel, immunofluorescence staining analysis of MAP2 displayed high fluorescent staining, indicating the successful isolation of hippocampal neurons from rats (Figure 2b). The absence of magnesium is a well-accepted stimulus for EP. Therefore, isolated hippocampal neurons were exposed to magnesium-free conditions. Interestingly, the downregulation of miR-30b-5p was detected in magnesium-free-treated hippocampal neurons (Figure 2c). However, the reduced expression of miR-30b-5p in hippocampal neurons as a consequence of magnesium-free induction was rescued after the cells were transfected with the miR-30b-5p mimic (Figure 2d). Magnesium-free treatment reduces the proliferation of hippocampal neurons while accelerating apoptosis. However, alterations in both hippocampal neuron proliferation and apoptosis were abrogated by the miR-30b-5p mimic (Figure 2e and f). Collectively, miR-30b-5p ectopic expression might protect hippocampal neurons from magnesium-free injuries.

### 3.3 miR-30b-5p overexpression restored the pathological injury and cell apoptosis in the hippocampal CA1 region

**Figure 3:**
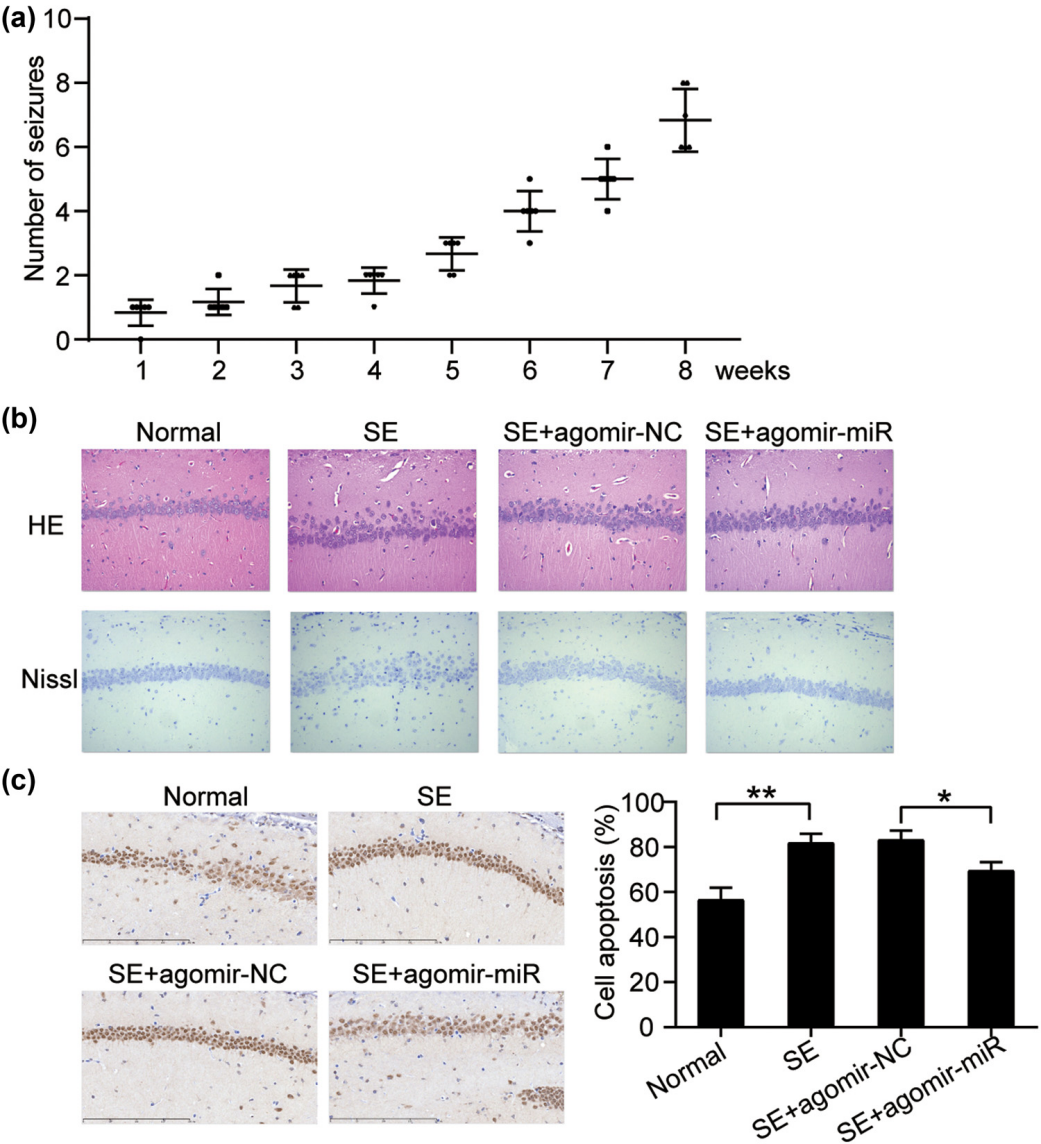
miR-30b-5p overexpression restored the pathological injury and cell apoptosis in hippocampal CA1 region. (a) Number of seizures of mice increases in the model group. (b) Pathological changes of hippocampal CA1 region were found in the model group after miR-30b-5p agomir or agomir negative control (agomir NC) was immediately injected via the tail vein. Hippocampal CA1 region using HE staining (400×) and Nissl’s staining. (c) TUNEL staining determining cell apoptosis rate in seizures. *P < 0.05, **P < 0.001. NC, negative control; HE, hematoxylin and eosin; SE, seizures.

In addition to the role of miR-30b-5p *in vitro*, we examined its function *in vivo*. First, we constructed a mouse model of EP by intraperitoneal injection of pilocarpine. Accumulating spontaneous seizures in mice were detected in the PE mouse model (Figure 3a). Furthermore, most mice presented with spontaneous recurrent seizures. These data suggest the successful establishment of an *in vivo* EP model. Next, agomir-30b-5p or agomir-NC was injected into the established mouse model, and hippocampal CA1 was isolated for the assessment of pathological alterations. As shown in Figure 3b, in the EP model, an increased number of Nissl bodies and serious pathological disorders were observed in the hippocampal CA1 area, while agomir-30b-5p introduction almost recovered the induced damage in the hippocampal CA1 area (Figure 3b). TUNEL apoptosis detection showed that hippocampal CA1 in mice suffering from seizures was characterized by high apoptosis, while agomir-30b-5p nullified accelerated apoptosis (Figure 3c). Therefore, miR-30b-5p may protect against induced disorders in the hippocampal CA1 region *in vivo*.

### 3.4 miR-30b-5p targets GRIN2A interfering with the magnesium-free condition that induces the growth inhibition of hippocampal neurons

**Figure 4:**
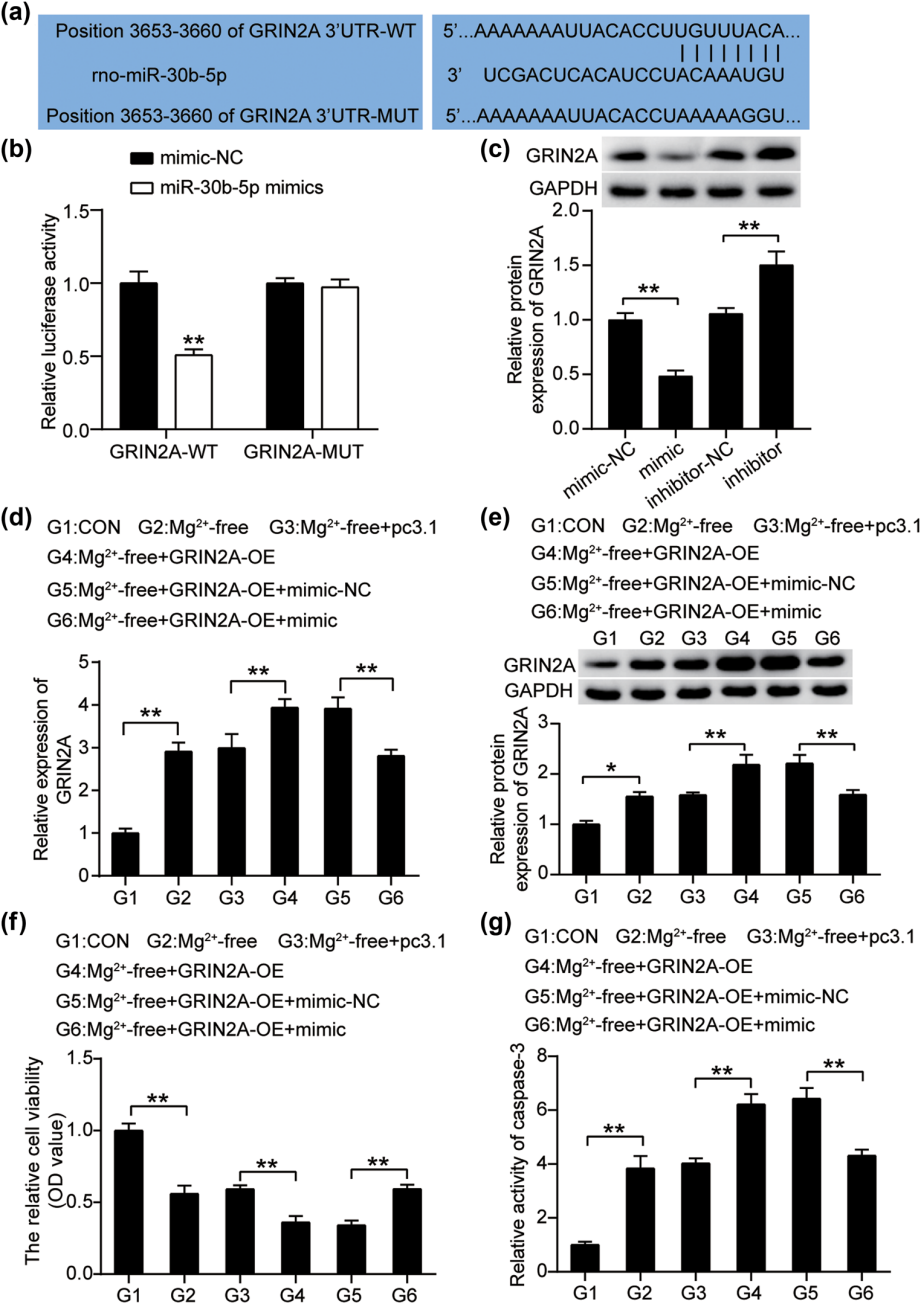
miR-30b-5p targets GRIN2A interfering with the magnesium-free condition that induces the growth inhibition of hippocampal neurons. (a) Gene structure of GRIN2A indicated the predicted target site of miR-30b-5p in its 3′-UTR. (b) Luciferase activity was measured in hippocampal neurons following co-transfecting with WT/MUT GRIN2A 3′-UTR plasmid and miR-30b-5p with the dual luciferase reporter assay. (c) Expression of GRIN2A protein in hippocampal neurons transfected with mimic-NC, miR-30b-5p mimic (mimic), inhibitor-NC, and miR-30b-5p inhibitor (inhibitor) was detected by western blots. (d) Expression of GRIN2A in hippocampal neurons transfected with pcDNA3.1-GRIN2A, pcDNA3.1, miR-30b-5p mimic, and miR-30b-5p mimic + pcDNA3.1-GRIN2A before magnesium-free treatment was investigated by RT-qPCR. (e) Western blotting analysis of GRIN2A expression in hippocampal neurons in the same groups as (d). (f) CCK8 assays determine the cell proliferation in the same groups as (d). (g) Caspase-3 activity measured in the same groups as (d). **P < 0.001. WT, wild type; MUT, mutant.

Consistent with the predicted interplay between GRIN2A and miR-30b-5p, Figure 4a reveals that miR-30b-5p shared interaction motifs with the 3′-UTR of GRIN2A. There was a conspicuous decrease in 3′-UTR GRIN2A-WT-derived luciferase reporter activity in hippocampal neurons transfected with the miR-30b-5p mimic, while no change was observed in 3′-UTR GRIN2A-MUT-driven activity (Figure 4b). Western blot analysis confirmed that the miR-30b-5p mimic suppressed GRIN2A expression, whereas the miR-30b-5p inhibitor enhanced GRIN2A expression (Figure 4c). To further investigate whether GRIN2A is important for the role of miR-30b-5p in EP, we co-transfected GRIN2A overexpressing vectors and miR-30b-5p mimic into hippocampal neurons before magnesium-free treatment. RT-qPCR analysis and western blotting demonstrated that the miR-30b-5p mimic abolished exogenous overexpression of GRIN2A (Figure 4d and e). Moreover, GRIN2A overexpression caused an extra induction in the proliferation of hippocampal neurons following magnesium-free treatment; however, the miR-30b-5p mimic almost restored the reduced proliferative capacity (Figure 4f). Undoubtedly, GRIN2A overexpression aggravated the proliferative defect caused by magnesium-free treatment, whereas the defect was abrogated by co-transfection with the miR-30b-5p mimic (Figure 4g). Collectively, these data suggest that miR-30b-5p targeting of GRIN2A hampers EP progression *in vitro*.

## 4 Discussion

The pathogenesis of EP involves the abnormal expression of various genes and transcripts [10]. Among these, miRNAs are implicated in synaptic plasticity, neuronal apoptosis, and neuroinflammation. Therefore, further elucidation of the role of miRNAs in EP may favor EP diagnosis and treatment [25]. In this study, we found that miR-30b-5p overexpression restored the reduced growth of hippocampal neurons caused by the magnesium-free condition. Subsequent investigation in EP model mice revealed that miR-30b-5p upregulation attenuated pathological injuries of hippocampal CA1 region of mice as well as declined the apoptosis of hippocampal CA1 region of mice. These findings suggest that targeting miR-30b-5p is a promising anti-EP method.

Magnesium-free conditions can induce recurrent spontaneous epileptic discharges in hippocampal neurons, which is similar to the electrophysiology of human EP [26]. During this process, various EP-related transcripts are activated. Therefore, we constructed an *in vitro* EP model to investigate the role of miR-30b-5p in EP. This miRNA is reported to be involved in diverse disorders such as diabetic retinopathy [27], myocardial infarction [28], and cancer [29,30]. However, its effect on cell proliferation and apoptosis is context- or tissue-dependent. For example, in breast cancer, miR-30b-5p increases the malignant behavior of tumor cells and acts as an oncomir [28]. miR-30b-5p was also shown to suppress the proliferation of cardiac fibroblasts and attenuate fibrogenesis post-myocardial infarction [31]. Our data demonstrated that the miR-30b-5p mimic promoted cellular proliferation and reduced apoptotic activity in hippocampal neurons exposed to magnesium-free conditions. Next, we dissected the role of miR-30b-5p in an EP mouse model. Consistent with the *in vitro* findings, we found that miR-30b-5p decreased apoptosis in the hippocampal CA1 region of EP mice, in addition to reversing the pathological changes in EP mice. Our findings suggest that miR-30b-5p exerts its anti-EP effect by increasing proliferation and impeding apoptosis in hippocampal neurons.

Generally, miRNAs post-transcriptionally edit mRNA and are involved in various pathophysiological processes. Based on our bioinformatic analysis, we verified that GRIN2A is a target of miR-30b-5p in hippocampal neurons. Previously, GRIN2A mutant was found to be associated with EP [17,32,33]. In our *in vitro* EP model, overexpression of GRIN2A led to extra suppression of proliferation in hippocampal neurons exposed to magnesium-free conditions as well as higher apoptosis compared with other groups, suggesting that GRIN2A overexpression can exacerbate EP status. The mechanical outcome demonstrated that the miR-30b-5p mimic abrogated the exogenous expression of GRIN2A in hippocampal neurons in the presence of magnesium-free conditions. Furthermore, the miR-30b-5p mimic abrogated the promotion of cell growth of GRIN2A overexpression in an *in vitro* EP model. Collectively, miR-30b-5p may attenuate the proliferation of hippocampal neurons in magnesium-free conditions by targeting GRIN2A.

However, this study had several limitations. First, the sample size was small and we only investigated the roles of miR-30b-5p and GRIN2A *in vitro* and *in vivo*. miRNAs can recognize different genes through seed-complementary sequences. Therefore, the complex regulatory network of miR-30b-5p in EP requires further investigation. The mTOR signaling [34], zinc signaling [35], and inflammation-associated signaling [36] are dysregulated during EP initiation and progression. Therefore, GRIN2A-mediated signaling pathways warrant further research.

Collectively, our data revealed that miR-30b-5p targeting GRIN2A promoted the proliferation and attenuated apoptosis of hippocampal neurons in an *in vitro* EP model. Furthermore, miR-30b-5p overexpression exerted anti-EP effects by suppressing apoptosis and pathological injuries in the hippocampal CA1 region in an EP mouse model. Our findings provide a promising approach for EP diagnosis and intervention.

## Abbreviations

DEGs: differentially expressed genes
EP: epilepsy
HE: hematoxylin and eosin
miRNAs: microRNAs
MUT: mutant
SE: status epilepticus
